# *N*-Methyl-d-Aspartate Receptor – Nitric Oxide Synthase Pathway in the Cortex of Nogo-A-Deficient Rats in Relation to Brain Laterality and Schizophrenia

**DOI:** 10.3389/fnbeh.2013.00090

**Published:** 2013-08-12

**Authors:** Zdena Krištofiková, Monika Vrajová, Jana Šírová, Karel Valeš, Tomáš Petrásek, Kai Schönig, Björn Tews, Martin Schwab, Dusan Bartsch, Aleš Stuchlík, Daniela Řípová

**Affiliations:** ^1^Prague Psychiatric Centre, Prague, Czech Republic; ^2^Institute of Physiology, Academy of Sciences, Prague, Czech Republic; ^3^Central Institute of Mental Health, Heidelberg University, Mannheim, Germany; ^4^German Cancer Research Centre, Heidelberg, Germany; ^5^Brain Research Institute, University of Zürich, Zürich, Switzerland

**Keywords:** brain laterality, animal model of schizophrenia, aging, myelin-associated protein, cortex

## Abstract

It has been suggested that Nogo-A, a myelin-associated protein, could play a role in the pathogenesis of schizophrenia and that Nogo-A-deficient rodents could serve as an animal model for schizophrenic symptoms. Since changes in brain laterality are typical of schizophrenia, we investigated whether Nogo-A-deficient rats showed any signs of disturbed asymmetry in cortical *N*-methyl-d-aspartate (NMDA) receptor–nitric oxide synthase (NOS) pathway, which is reported as dysfunctional in schizophrenia. In particular, we measured separately in the right and left hemisphere of young and old Nogo-A-deficient male rats the expression of NMDA receptor subunits (NR1, NR2A, and NR2B in the frontal cortex) and activities of NOS isoforms [neuronal (nNOS), endothelial (eNOS), and inducible (iNOS) in the parietal cortex]. In young controls, we observed right/left asymmetry of iNOS activity and three positive correlations (between NR1 in the left and NR2B laterality, between NR2B in the right and left sides, and between NR1 in the right side and nNOS laterality). In old controls, we found bilateral decreases in NR1, an increase in NR2B in the right side, and two changes in correlations in the NR1–nNOS pathway. In young Nogo-A-deficient rats, we observed an increase in iNOS activity in the left hemisphere and two changes in correlations in NR1–nNOS and NR2A–eNOS, compared to young controls. Finally, we revealed in old Nogo-A-deficient animals, bilateral decreases in NR1 and one change in correlation between eNOS–iNOS, compared to old controls. Although some findings from schizophrenic brains did not manifest in Nogo-A-deficient rats (e.g., no alterations in NR2B), others did (e.g., alterations demonstrating accelerated aging in young but not old animals, those occurring exclusively in the right hemisphere in young and old animals and those suggesting abnormal frontoparietal cortical interactions in young animals).

## Introduction

It has been suggested that the lateralization of the human brain, i.e., differences between the right (R) and left (L) hemisphere, underlies hemispheric specialization that can also be observed biochemically. Biochemical laterality appears to be a basis of volumetric or functional asymmetry; however, direct relationships among them are still unclear. Brain asymmetry is moderately changed during normal aging and more markedly in neurodegenerative disorders like Alzheimer disease or in neurodevelopmental diseases like schizophrenia (for a review, see Toga and Thompson, 2003). Our previous studies suggested a high sensitivity of lateral correlation analyses in revealing subtle links among the biochemical pathways. We therefore recommended their use in animal models of various diseases that are accompanied by alterations in brain asymmetry. Knowledge of disease-mediated changes occurring in the human R and L hemisphere separately as well as that of brain lateralization of applied control animals (there are differences between mice and rats and also among various rodent strains) is necessary in validating animal models in this way (Krištofiková et al., 2004, 2008, 2010). Moreover, we have repeatedly found a very similar degree of brain asymmetry on a biochemical level in the hippocampus and cortex, two of the most asymmetrical brain regions (Krištofiková et al., 2010).

The biological basis of schizophrenia is still unknown, but many studies have indicated that oligodendroglial dysfunction with subsequent abnormalities in myelin maintenance and repair can contribute to the pathogenesis of this major psychiatric disorder (Davis et al., 2003). Myelin-associated proteins and especially the myelin-associated glycoprotein Nogo-A (encoded by the *RTN4* gene located on chromosome 2p16.1 and expressed in oligodendrocytes or neurons) and its glycosylphosphatidylinositol-linked Nogo-66 receptor 1 (encoded by the *RTN4R* gene located on chromosome 22q11 and expressed on the surface of neurons or fibroblasts) are important for cellular remodeling. Nogo-A inhibits neurite outgrowth, and alters endothelial and smooth muscle cell migration and adhesion (Dodd et al., 2005). Postmortem or genetic studies report an involvement of Nogo-A (Coon et al., 1998; Shaw et al., 1998; Novak et al., 2002; Tan et al., 2005; Novak and Tallerico, 2006; Jitoku et al., 2011) or its receptor (Sinibaldi et al., 2004; Hsu et al., 2007; Budel et al., 2008) in schizophrenia; however, changes such as varying Nogo-A mRNA levels in the autoptic frontal cortices of psychotic patients are not marked, in contrast to those in Nogo-C mRNA (Novak and Tallerico, 2006).

On the other hand, Nogo-A knock-out animals have some intermediate phenotypes resembling disorders of neurodevelopmental origin, such as schizophrenia. Deletion of Nogo-A increases the motility of embryonic forebrain-derived neurospheres and decreases the accumulation of migrating neuronal precursors in the newborn cortex (Mathis et al., 2010). However, the changes in the mature brain tissue are subtle (e.g., there is significantly increased neurite outgrowth in spinal cord extracts, but no changes in gross brain anatomy, extracellular matrix markers, glial markers, and oligodendrocytes morphology; see Simonen et al., 2003). Nevertheless, young adult Nogo-A knock-out rodents show schizophrenia-like abnormalities in behavioral tests (e.g., deficient sensorimotor gating, disrupted latent inhibition, perseverative behavior, and increased sensitivity to the locomotor stimulating effects of amphetamine) and in neurochemical analysis (e.g., altered monoaminergic transmitter levels and changes in dopamine D2 receptor levels in striatal and limbic regions) (see Willi et al., 2010; Tews et al., 2013). A recent study reviewed data from various animals displaying deficient Nogo-A and/or its receptor, and suggested that schizophrenia-like abnormalities were based on deregulated brain connectivity (Willi and Schwab, 2013).

Our previous studies focused on lateral alterations in the levels of the *N*-methyl-d-aspartate receptor (NMDAR) subunit NR1 and nitric oxide synthase (NOS) in postmortem brains of schizophrenic patients compared to non-psychotic controls. We found no pronounced laterality in mRNA/protein expression of NR1 in the hippocampi of the controls, but a significant drop in NR1 expression in the L side of psychotic men (Vrajová et al., 2010). Regarding the activities or expressions of the NOS isoforms, we observed no asymmetry in neuronal (nNOS), marked R/L asymmetry in endothelial (eNOS, dominance in the R side was observed especially in its activity) and marked L/R asymmetry in inducible (iNOS, dominance in the L side was found exclusively in its activity) isoforms in the hippocampi of controls. In people with schizophrenia, we found more marked increases in nNOS/iNOS activity (but not in their expression) in the R than L hemisphere (Krištofiková et al., 2008).

*N*-methyl-d-aspartate receptors are associated with NOS isoforms through a postsynaptic density protein and a stimulation of the receptors activates synthesis of nitric oxide through the nNOS/eNOS/iNOS isoforms. Functional interactions between NR1 subunits and nNOS (as well as between NR2A/2B and the other NOS enzymes) were proposed (e.g., Kamida et al., 2007). In the brain of schizophrenic patients, there was a drop in the NR1 subunit mRNA level in the dentate gyrus (Gao et al., 2000), but no significant changes in NR1 protein amounts in the superior temporal cortex (Nudmamud-Thanoi and Reynolds, 2004). For the NR2A subunit, no changes in mRNA expression in the anterior cingulate cortex (Woo et al., 2004) or in the hippocampal subregions (Gao et al., 2000) were observed. On the other hand, alterations in the expression of the NR2B subunit were clearly apparent in schizophrenia. Namely, increases in NR2B mRNA concentrations in hippocampal CA2 and CA3 (Gao et al., 2000), and the elevation of NR2B protein levels in the thalamus (Clinton and Meador-Woodruff, 2004; Clinton et al., 2006) were found. Some studies have reported associations between the NR2B gene and schizophrenia (e.g., Li and He, 2007), supporting a role of NR2B gene polymorphisms in language lateralization (Ocklenburg et al., 2011). Moreover, changes in the postsynaptic density protein, e.g., in the dorsomedial thalamus (Clinton and Meador-Woodruff, 2004; Clinton et al., 2006), support a dysfunction of the NMDAR-NOS pathway in schizophrenia.

The aims of the study were to determine the expression of the NR1/NR2A/NR2B subunits and activities of nNOS/eNOS/iNOS isoforms separately in the R and L cortex of young and old Nogo-A-deficient rats, as well as to validate the animal model of schizophrenia by evaluating alterations in brain laterality.

## Materials and Methods

### Animals

Nogo-A-deficient transgenic animals were generated using microRNA interference (for more details, see Tews et al., 2013). Experiments were performed on young (3–5 months) and old (10–12 months) Nogo-A-deficient Sprague-Dawley rats or age-matched controls. All rats were housed in groups of two under a 12-h light/dark cycle. Food (standard pellet diet) and water were available *ad libitum*. All experiments were carried out in accordance with the Animal Protection Law 246/1992 and the Regulation 419/2012 (Czech Republic) concerning the use of experimental animals, based on NIH guidelines and EU directives (2010/63/EU).

### Tissue sampling

Rats were sacrificed by cervical dislocation, decapitated, and the brains rapidly removed. The frontal and parietal cortices were dissected, weighed, packed in aluminum foil, and frozen at −40°C until assayed (no more than 2 weeks later).

### Expression of the NMDAR subunits NR1, NR2A, and NR2B by western blotting

The frontal cortices were homogenized in 1.0 mL of lysis buffer (320 mM sucrose; 10 mM Tris, pH 7.4; 0.2 mM EDTA; 2 mM PMSF; 1 mM 2-mercaptoethanol; and a cocktail of protease inhibitors, Sigma). Crude synaptosomal (P_2_) fractions were isolated from homogenates and resuspended in a loading buffer (63 mM Tris; 10% glycerol; 2% SDS; 5% 2-mercaptoethanol; and 0.01% bromophenol blue). The protein concentration was determined by the Bradford method using bovine serum albumin (BSA) as the standard (Bio-Rad, USA). The resuspended material was subjected to electrophoresis in the 7.5% polyacrylamide gel (Criterion Cell, Bio-Rad, USA), followed by electroblotting in the Criterion blotter (Bio-Rad, USA). Non-specific binding was blocked with 3% BSA dissolved in TBS-T buffer. Blots were incubated overnight with anti-NMDAR1 (1:100; Millipore, USA) or for 2 h with anti-NMDAR2A/2B (1:500; Millipore, USA) primary antibody. For loading control, blots were treated with an anti-α-tubulin antibody (1:1000; Exbio, CZ) for 1 h. Then, the blots were washed in TBS-T buffer and incubated for 1 h with a horseradish peroxidase-conjugated secondary antibody (1:3000; Dako, Denmark). Detections were performed with a chemiluminescent substrate (Pierce, USA) and evaluated by the Gel Doc Analysis system (Bio-Rad, USA).

### Activities of nNOS, eNOS, and iNOS

The parietal cortices were homogenized (1:10) in homogenization buffer (1 mM EGTA, 1 mM dithiothreitol, 20 mM HEPES, 0.32 M sucrose, 14.6 μM pepstatin, and 21 μM leupeptin, pH = 7.4) and the resulting homogenates centrifuged at 1200 *g* for 10 min at 4°C. Supernatants were added to the reaction buffer [homogenization buffer containing also 200 μM β-nicotinamide adenine dinucleotide phosphate, 50 μM tetrahydrobiopterin, and 4.6 μM [14C]arginine (PerkinElmer)] and incubated for 30 min at 37°C. Some samples also contained 1 μM CaCl_2_ (nNOS and eNOS) and specific inhibitors (1 mM spermidine for nNOS, 190 μM Nω-nitro-l-arginine methyl ester for nNOS/eNOS, and 1 mM aminoguanidine for iNOS, all from Sigma). Final protein concentrations determined by the Bradford method equaled 0.5 mg/mL in all incubation mixtures. The reaction was terminated by adding the stop buffer (30 mM HEPES, 3 mM EDTA, pH = 5.5) and by rapid cooling. DOWEX 50WX8-200 (Sigma) was used to separate citrulline from arginine, in accordance with our previous study (Krištofiková et al., 2008).

### Statistical analysis

The BMDP statistical software (non-parametric Kruskal–Wallis test for global analysis and Mann–Whitney–Wilcoxon test for pairwise comparisons) or SigmaStat statistical software (Spearman rank order correlation) were used. Differences between correlation coefficients were evaluated using a Rao test based on the Fisher *Z*-transformation. The index of laterality [(L − R)/(L + R)] was calculated to estimate differences between the R and L sides. The index is limited to zero when all the values are not lateralized (marked asymmetry was defined in this study by indexes>±0.100) or when the numbers of markedly R/L (dominance of the R side) and L/R (dominance of the L side) animals are approximately equal. Data in the tables are presented as the means ± SEM.

## Results

Results in Table 1 demonstrate no pronounced laterality in the expression of the NR1, NR2A, and NR2B subunits in the frontal cortex of young Sprague-Dawley male rats. In old compared to young controls, we observed a significant bilateral reduction in NR1 expression (to 94% in the R and to 89% in the L side, both compared to the corresponding hemisphere of the young controls), no changes in NR2A expression and a significant increase in NR2B expression (to 108%) only in the R side of the brain. We also did not find significant changes in NMDAR subunit expression in young Nogo-A-deficient rats compared to the age-matched control group. In old Nogo-A-deficient rats, we observed bilateral increases in NR1 subunit levels (to 106% in the R and to 113% in the L side, both compared to the corresponding hemisphere of the old controls), but no changes in NR2A or NR2B subunit expression compared to the old control group.

**Table 1:** **NR1, NR2A, and NR2B subunit expression**.

Groups	*n*	R	L	(L − R)/(L + R)
**NR1**				
Young controls	10	1.031 ± 0.007	1.036 ± 0.008	+0.002 ± 0.004
Young NOGO	9	1.044 ± 0.007	1.055 ± 0.008	+0.005 ± 0.006
Old controls	9	0.972 ± 0.018**	0.924 ± 0.035**	−0.028 ± 0.017
Old NOGO	8	1.032 ± 0.023+	1.047 ± 0.021++	+0.007 ± 0.013
Kruskal–Wallis		χ^2^(3) = 12.36	χ^2^(3) = 18.48	χ^2^(3) = 4.45
		*p* = 0.0062	*p* = 0.0004	*p* = 0.2169
**NR2A**				
Young controls	10	1.198 ± 0.007	1.198 ± 0.011	+0.000 ± 0.003
Young NOGO	9	1.198 ± 0.020	1.193 ± 0.017	−0.002 ± 0.007
Old controls	9	1.188 ± 0.015	1.177 ± 0.018	−0.007 ± 0.005
Old NOGO	8	1.214 ± 0.015	1.217 ± 0.015	+0.010 ± 0.004
Kruskal–Wallis		χ^2^(3) = 1.27	χ^2^(3) = 2.52	χ^2^(3) = 6.34
		*p* = 0.7367	*p* = 0.4719	*p* = 0.0963
**NR2B**				
Young controls	10	0.876 ± 0.017	0.871 ± 0.012	−0.002 ± 0.004
Young NOGO	9	0.897 ± 0.010	0.900 ± 0.020	+0.001 ± 0.009
Old controls	9	0.945 ± 0.013**	0.887 ± 0.030	−0.034 ± 0.018
Old NOGO	8	0.902 ± 0.033	0.864 ± 0.058	−0.028 ± 0.030
Kruskal–Wallis		χ^2^(3) = 9.01	χ^2^(3) = 2.20	χ^2^(3) = 4.32
		*p* = 0.0291	*p* = 0.5312	*p* = 0.2288

*Means ± SEM, NOGO – Nogo-A-deficient rats*.

Mann–Whitney–Wilcoxon test was calculated with respect to young (**p < 0.010) or old (+*p* < 0.050, ++*p* < 0.010) controls.

Table 2 shows no pronounced asymmetry in nNOS and eNOS activities, but a strong R/L asymmetry of iNOS activity in the parietal cortex of young controls. In the old controls, no significant differences in the activities of all synthases were found when compared to corresponding hemispheres of young controls. However, bilateral increases in nNOS (to 117 and 123% in the R and L sides, respectively) and bilateral decreases in eNOS (to 71 and 82% in the R and L sides, respectively) were observed. Moreover, the shift from the marked R/L to L/R asymmetry of iNOS was striking (see the drop to 29% in the R and the increase to 245% in the L side, Mann–Whitney–Wilcoxon test for indexes of laterality between young and old rats; *p* = 0.1350). In young Nogo-A-deficient rats, there were no changes in nNOS or eNOS activities compared to age-matched controls. On the other hand, the results of the Mann–Whitney–Wilcoxon tests supported the significant increase of iNOS activity to 351% in the L side of Nogo-A-deficient rats despite the insignificant results of the global Kruskal–Wallis test. We did not observe pronounced alterations in old Nogo-A-deficient rats compared to age-matched controls; however, the shift from L/R asymmetry in old controls to R/L asymmetry in old Nogo-A-deficient rats regarding iNOS activity was again striking (i.e., the increase to 290% in the R but a drop to 47% in the L side, Mann–Whitney–Wilcoxon test for indexes of laterality: *p* = 0.2808).

**Table 2:** **The activities of nNOS, eNOS, and iNOS**.

Groups	*n*	R	L	(L − R)/(L + R)
**nNOS**				
Young controls	10	1141.0 ± 101.2	1146.6 ± 86.0	+0.007 ± 0.039
Young NOGO	9	1125.4 ± 39.9	1100.8 ± 75.2	−0.018 ± 0.025
Old controls	9	1335.4 ± 153.4	1413.3 ± 167.5	+0.045 ± 0.059
Old NOGO	8	1252.4 ± 175.3	1220.3 ± 161.5	+0.000 ± 0.055
Kruskal–Wallis		χ^2^(3) = 4.66	χ^2^(3) = 4.16	χ^2^(3) = 1.16
		*p* = 0.1983	*p* = 0.2442	*p* = 0.7628
**eNOS**				
Young controls	10	829.7 ± 109.8	729.9 ± 138.8	−0.086 ± 0.079
Young NOGO	9	830.6 ± 146.4	788.2 ± 145.2	−0.034 ± 0.069
Old controls	9	586.9 ± 98.7	600.6 ± 102.6	+0.028 ± 0.051
Old NOGO	8	558.7 ± 107.3	450.8 ± 91.1	−0.053 ± 0.140
Kruskal–Wallis		χ^2^(3) = 3.60	χ^2^(3) = 2.67	χ^2^(3) = 0.56
		*p* = 0.3077	*p* = 0.4462	*p* = 0.9046
**iNOS**				
Young controls	10	34.4 ± 13.4	12.5 ± 7.2	−0.405 ± 0.209
Young NOGO	9	41.0 ± 17.5	43.9 ± 13.5*	+0.110 ± 0.266
Old controls	9	10.0 ± 5.2	30.6 ± 13.9	+0.114 ± 0.309
Old NOGO	8	29.0 ± 9.5	14.5 ± 7.0	−0.281 ± 0.136
Kruskal–Wallis		χ^2^(3) = 3.65	χ^2^(3) = 4.98	χ^2^(3) = 4.33
		*p* = 0.3018	*p* = 0.1732	*p* = 0.2276

*Means ± SEM, NOGO – Nogo-A-deficient rats*.

Mann–Whitney–Wilcoxon test was calculated with respect to young controls (*p < 0.050).

Results of the correlation analyses performed on young control rats (data are shown in Tables 3 and 4) supported links: (i) among the subunits of NMDAR (after a Bonferroni correction, two significant positive correlations were found, between NR1 in the L side and laterality of NR2B, and between NR2B in the R and L sides); and (ii) among the subunits and synthases (one significant positive correlation was found after a Bonferroni correction between NR1 in the R side and laterality of nNOS). A comparison of correlations from 10 young and 9 old control rats is presented in Table 3. The table shows all significant results with regard to the Rao test; however, only two significant age-related changes were found after the Bonferroni correction. First, there was a significant shift from a positive correlation in young to a negative correlation in old controls between the NR1 subunit in the R side and nNOS laterality. Second, there was a significant shift from a negative correlation in young to a positive correlation in old controls between the NR1 subunit in the L side and nNOS activity in the R side. Finally, a comparison of the correlation data from 9 young Nogo-A-deficient rats (and 10 age-matched controls) and 8 old Nogo-A-deficient rats (and 9 corresponding controls) is given in Table 4. As in Table 3, all significant results based on the Rao test are presented. In young Nogo-A-deficient rats, when compared to the age-matched controls, we observed two significant changes after a Bonferroni correction. First, there was a shift from a negative correlation in control to a positive correlation in Nogo-A-deficient rats between the NR1 subunit in the L side and nNOS activity in the R side (similar to that seen with normal aging). Second, there was a significant shift from a negative correlation in control to a positive correlation in Nogo-A-deficient rats between NR2A and eNOS, both in the R side (again similar to that observed in normal aging; however, this was not statistically significant in relation to aging: *p* = 0.0749, data not shown). Moreover, we found a marked shift from an insignificant correlation in old controls to a significantly positive correlation in old Nogo-A-deficient rats between the activities of eNOS and iNOS in the R side.

**Table 3:** **The result of correlation analysis performed on young and old control rats**.

Parameters	Young controls		Old controls		Rao *p*
	CC	*p*	CC	*p*	
NR1 R × nNOS laterality	+0.830	<0.001*	−0.762	0.012	<0.001*
NR1 R × iNOS L	−0.350	0.309	+0.714	0.025	0.023
NR1 L × NR2B R	−0.612	0.054	+0.450	0.204	0.032
NR1 L × NR2B laterality	+0.842	<0.001*	−0.133	0.709	0.014
NR1 L × nNOS R	−0.770	0.007	+0.583	0.087	0.002*
NR1 L × nNOS L	−0.648	0.038	+0.433	0.223	0.026
NR1 laterality × nNOS L	−0.770	0.007	+0.567	0.099	0.003
NR2A R × eNOS R	−0.467	0.160	+0.450	0.204	0.075
NR2A R × iNOS laterality	+0.720	0.016	−0.443	0.204	0.013
NR2A L × eNOS R	−0.491	0.137	+0.633	0.058	0.021
NR2A L × iNOS laterality	+0.603	0.060	−0.523	0.138	0.022
NR2A laterality × nNOS laterality	−0.382	0.258	+0.686	0.036	0.026
NR2B R × NR2B L	+0.903	<0.001*	+0.467	0.186	0.078
NR2B L × NR2B laterality	−0.430	0.199	+0.683	0.036	0.020
nNOS R × iNOS R	−0.717	0.016	+0.365	0.308	0.021

Spearman rank order correlation was first performed on young and old controls, and subsequently particular correlation coefficients (CC) were compared using the Rao test. Finally, *Bonferroni correction was performed.

**Table 4:** **The results of correlation analysis performed on Nogo-A-deficient rats and corresponding controls**.

Parameters	Groups	Controls		NOGO		Rao *p*
		CC	*p*	CC	*p*	
**SUBUNITS VS. SUBUNITS**						
NR1 R × NR2A L	Young	−0.636	0.043	+0.483	0.169	0.022
NR1 R × NR2B R	Young	−0.442	0.185	+0.833	0.002*	0.003
NR1 R × NR2B L	Young	−0.491	0.137	+0.667	0.043	0.016
NR1 L × NR2B laterality	Young	+0.842	<0.001*	+0.017	0.948	0.030
NR2A R × NR2B L	Old	−0.333	0.356	+0.667	0.059	0.046
NR2B L × NR2B laterality	Young	−0.430	0.199	+0.617	0.067	0.034
**SYNTHASES VS. SYNTHASES**						
nNOS R × nNOS laterality	Young	−0.697	0.022	+0.393	0.264	0.022
nNOS R × iNOS R	Young	−0.717	0.016	+0.218	0.550	0.044
nNOS L × iNOS R	Young	−0.498	0.126	+0.628	0.058	0.021
eNOS R × iNOS R	Old	−0.091	0.809	+0.976	<0.001*	<0.001*
iNOS R × iNOS L	Old	−0.343	0.331	+0.832	0.005	0.007
**SUBUNITS VS. SYNTHASES**						
NR1 R × nNOS laterality	Young	+0.830	<0.001*	−0.402	0.264	0.004
NR1 R × iNOS laterality	Old	+0.541	0.124	−0.756	0.021	0.006
NR1 L × nNOS R	Young	−0.770	0.007	+0.583	0.087	0.002*
NR1 L × nNOS L	Young	−0.648	0.038	+0.400	0.264	0.032
NR1 laterality × nNOS L	Young	−0.770	0.007	+0.133	0.709	0.038
NR1 laterality × iNOS R	Old	+0.785	0.009	−0.439	0.206	0.008
NR1 laterality × iNOS laterality	Old	−0.674	0.043	+0.708	0.037	0.003
NR2A R × eNOS R	Young	−0.467	0.160	+0.883	<0.001*	<0.001*
NR2A R × eNOS L	Young	−0.358	0.292	+0.683	0.036	0.030
NR2A R × iNOS L	Young	+0.798	0.004	−0.167	0.643	0.023
NR2A R × iNOS laterality	Young	+0.720	0.016	−0.294	0.407	0.030
NR2A L × eNOS R	Young	−0.491	0.137	+0.600	0.077	0.027
NR2A L × nNOS R	Old	−0.050	0.878	−0.857	0.002*	0.033
NR2B laterality × nNOS L	Old	−0.683	0.036	+0.311	0.423	0.045

Spearman rank order correlation was first performed on Nogo-A-deficient rats and on corresponding controls. Particular correlation coefficients (CC) were then compared by the Rao test. Finally, a *Bonferroni correction was performed.

## Discussion

### NMDAR subunits and NOS isoforms in the cortex of control Sprague-Dawley rats and effects of normal aging

Our results demonstrated no pronounced differences in NR1, NR2A, and NR2B expression between the R and L sides of the frontal cortex in young adult Sprague-Dawley male rats (Table 1). Moreover, no individual young markedly lateralized (defined by an index of laterality greater than ±0.100) rat was identified. No asymmetrical differences in nNOS or eNOS activity were observed globally; however, in contrast to the NMDAR subunits, we found some lateralized individuals [namely, one-third of animals displayed marked R/L asymmetry of globally unlateralized nNOS/iNOS, one-third presented opposite L/R laterality, and only one-third was unlateralized, in accordance with our previous study (Krištofiková et al., 2010)]. On the other hand, young Sprague-Dawley males displayed pronounced R/L laterality in iNOS activity, similar to Wistar and Long Evans strains (Krištofiková et al., 2010). These results could be hypothetically interpreted as a brain laterality based on asymmetries in expression/activity of various enzymes, receptors or transporters (see Krištofiková et al., 2004, 2010), but not in NMDAR subunit expression. Our correlation analysis suggesting possible asymmetric links between NR1 and NR2B (Table 3, the positive correlation between NR1 in the L side and NR2B laterality and between NR2B in the R and L sides) and between NR1 and nNOS (Table 3, the positive correlation between NR1 in the R side and nNOS laterality) thus could reflect complicated regulatory mechanisms occurring between the two hemispheres, rather than just L-R lateralization. However, the situation may not be so simple. It is known that NR2B expression is equal in homogenates, but asymmetrical in synaptic fractions isolated separately from the R and L mouse hippocampus (L/R dominance; Kawakami et al., 2003). Thus, it is suggested that possible lateralization of NMDAR subunits could be associated with their allocations at the synaptic level.

In relation to normal aging, we observed bilateral decreases in NR1, no alterations in NR2A and a significant increase in NR2B exclusively in the R side of the frontal cortex of old compared to young control rats (Table 1). Although many studies have reported age-related decreases in the expression of NMDAR subunits in the rat/mouse cortex or hippocampus (e.g., Magnusson et al., 2002), our data supported it only in the case of the NR1 subunit. The discrepancy observed between NR2A and NR2B expression could be explained by their transient moderate increases in 10-month-old animals followed by significant drops in 30-month-old animals (Magnusson et al., 2002). Similarly, we did not find significant age-related changes in the activities of NOS isoforms in the parietal cortex (Table 2). However, there were moderate changes in old control rats that were very similar to those in Wistar/Long Evans strains (the increases in nNOS/iNOS especially in the L side and the bilateral decreases in eNOS; Krištofiková et al., 2010). Therefore, it appears that our old Sprague-Dawley controls were still relatively young to manifest pronounced age-related changes in their NMDAR subunits expression, except NR1.

Age-related changes in human brain laterality are based on a higher vulnerability of the L, i.e., dominant in the majority of cases, than of the R hemisphere to aging processes (Toga and Thompson, 2003). Previously, we noted similar asymmetrical changes in rat brains; however, they were subtle (although significant) and difficult to detect, even in very old animals (Krištofiková et al., 2010). In this study, although we observed a significant increase in NR2B expression exclusively in the R side, the statistical analysis using corresponding indexes of laterality did not support significant changes in brain asymmetry (Table 1, Kruskal–Wallis test: *p* = 0.2288; Mann–Whitney–Wilcoxon between the two control groups: *p* = 0.0864). Likewise, although the changes in iNOS activity of old Sprague-Dawley rats were similar to those of old Wistar/Long Evans rats (i.e., the drop in the R and the increase in the L side; Krištofiková et al., 2010), the statistical analysis based on corresponding indexes of laterality did not support significant changes again (Table 2, Kruskal–Wallis test: *p* = 0.2276; Mann–Whitney–Wilcoxon between the two control groups: *p* = 0.1350). Therefore, we believe that our results indicating significant asymmetrical alterations in the NR1–nNOS pathway during aging (Table 3, two significant shifts: from a positive to a negative correlation between NR1 in the R side and nNOS laterality; and from a negative to a positive correlation between NR1 in the L and nNOS in the R side) should be interpreted as normal aging-evoked disruption of regulatory mechanisms occurring between the two hemispheres rather than a higher impairment of the L than R hemisphere seen in very old animals (Krištofiková et al., 2010).

### Changes in Nogo-A-deficient rats and a validity of the animal model of schizophrenia

If we compare our results with those obtained from the autoptic brain tissue of patients with schizophrenia, we can observe that neither young nor old Nogo-A-deficient rats displayed cortical increases in NR2B expression and nNOS activity, especially the latter in the R side, compared to age-matched controls. On the other hand, some findings support a validity of this animal model of schizophrenia.

#### Accelerated aging in young but not old Nogo-A-deficient rats

If we compare young Nogo-A-deficient rats with age-matched controls, there is a significant increase in iNOS activity in the L side (Table 2) and two significant correlation changes (Table 4, the shifts from negative to positive correlations between NR1 in the L and nNOS in the R side and between NR2A and eNOS in the R side). Since these alterations in the cortical NMDAR–NOS pathway of young Nogo-A-deficient rats are very similar to those of old controls (despite the changes being only borderline significant in old controls), the data could be interpreted as accelerated aging of the young genetically modified animals. On the contrary, old Nogo-A-deficient rats did not seem to be the model of accelerated aging. First, we did not observe progressive changes in genetically modified animals. For example, we did not find in old Nogo-A-deficient rats the above-mentioned increase in iNOS in the L side (Table 2) or the alterations in the NR1–nNOS and NR2A-eNOS pathways (data not shown, *p* = 0.811 and 0.741, respectively). Second, the significant changes observed in old Nogo-A-deficient rats [i.e., the bilateral increases in NR1 (Table 1) and the shift from a negative to positive correlation between eNOS and iNOS in the R side (Table 4)] were opposite to those involved in normal aging.

It is well known that schizophrenia and Alzheimer disease are both accompanied by progressive cognitive decline and that some of the changes in brain biochemistry associated with synaptic functions and apoptosis are very similar (Krištofiková et al., 2008; Tang et al., 2009). For instance, the activities of NOS isoforms in the hippocampi are increased in schizophrenia and Alzheimer disease; however, they are more marked in the R side of psychotic patients and in the L side of people with dementia (Krištofiková et al., 2008). The identical changes in the NMDAR–NOS pathway occurring in the cortex of young Nogo-A-deficient rats and old controls support the hypothesis that schizophrenia is a syndrome of accelerated aging (e.g., Kirpatrick et al., 2008). On the other hand, differences between young and old Nogo-A-deficient rats agree well with studies reporting similar changes in the frontal cortex of patients at early stages of schizophrenia and older controls, but totally different changes in older psychotic patients at later stages of the disease (Tang et al., 2009). Our data are also consistent with non-progressive changes in the cortex in schizophrenia during aging, showing that pathological processes occur in a relatively limited period of time around the onset of illness (Kubota et al., 2011). We believe that the simple age-dependent progression of changes in the pathways related to synaptic functions or apoptosis should be a characteristic sign of neurodegenerative Alzheimer disease, which incidentally is associated with Nogo-A overexpression in senile plaques (Gil et al., 2006), but not of neurodevelopmental schizophrenia. Moreover, we postulate that evaluations of new models of neurodevelopmental diseases should be performed in early adolescent as well as late senescent animals to distinguish among early and late developmental changes (see also Rapoport et al., 2005).

#### Alterations in the NMDAR-NOS pathway occurring in the R hemisphere

As previously described, the increases in nNOS/iNOS activity are more marked in the R than L hippocampus of patients with schizophrenia (Krištofiková et al., 2008). Although the data from Nogo-A-deficient rats did not directly indicate more changes in the NMDAR–NOS pathway in the R side [see the increase in iNOS activity in the L side of young rats (Table 2) and the bilateral elevation of NR1 expression in old rats (Table 1)], our correlation analysis results focused attention on the R side (Table 4). First, neither young nor old Nogo-A-deficient rats displayed significant changes exclusively in the L side. Second, we discovered three statistically significant changes, one of which involved both hemispheres (the shift from negative to positive correlation between NR1 in the L and nNOS in the R side of young Nogo-A-deficient rats, which could be interpreted as a disruption of the regulatory mechanisms between the R and L sides in the NR1–nNOS pathway, similar to that seen in normal aging) and the remaining two were exclusively associated with the R side (the shifts from mildly negative to markedly positive correlations between NR2A and eNOS in young animals and between eNOS and iNOS in old animals). For a comparison, no corresponding changes were found in the L side (data not shown, Rao test for young animals: *p* = 0.224, that for old animals: *p* = 0.603).

With regard to the R hemisphere, data obtained from young Nogo-A-deficient rats without marked changes in NR2A expression or eNOS activity could be interpreted as an increased cooperation between NR2A and eNOS. It is important to note that NR2A overexpression has been found, e.g., in the prefrontal cortex of rats reared in isolation, i.e., in animals exhibiting several characteristics seen in schizophrenia (Turnock-Jones et al., 2009). Data from old Nogo-A-deficient rats without pronounced changes in eNOS and iNOS activities could be similarly interpreted as an increased cooperation between eNOS and iNOS. This is supported by studies reporting a stimulation of iNOS transcription *in vivo* by activated eNOS (Connelly et al., 2005). Our results thus indicate the enhanced role of eNOS especially in the R cortex of young or old Nogo-A-deficient rats.

#### Abnormal frontoparietal cortex interactions

It is well known that the frontoparietal cortical network for rapid visual information processing requires working memory. It is suggested that this network in the R side is specialized for sustained attention and in the L side, for phonological loop component of working memory. In patients with schizophrenia, data suggest prefrontal-parietal functional disconnections, particularly prefrontal dissociation and abnormal prefrontal–parietal cortical interaction, during working memory processing (Kim et al., 2003). In Nogo-A-deficient young and old rats, we did not find changes in correlations among particular NMDAR subunits, suggesting a possible prefrontal dissociation (Table 4). However, significant alterations in correlations between NMDAR subunits in the frontal cortex and NOS isoforms in the parietal cortex could indicate abnormal frontoparietal interactions. After a Bonferroni correction, there were two corresponding alterations only in young Nogo-A-deficient rats (the shifts from negative to positive correlations between NR1 in the L and nNOS in the R side and between NR2A and eNOS in the R side, see Table 4). Although we did not find similar significant changes in old genetically modified animals, some results displayed borderline significance here (e.g., correlations between NR1 in the R side and iNOS laterality, between NR1 laterality and iNOS in the R side and between NR1 laterality and iNOS laterality, see Table 4). Thus, possible abnormal frontoparietal cortex interactions should not be excluded in young as well as old Nogo-A-deficient rats.

## Conflict of Interest Statement

The authors declare that the research was conducted in the absence of any commercial or financial relationships that could be construed as a potential conflict of interest.
